# A Study of Variation in the Major Phenolic Acid Components of Dandelions Across Different Regions, and the Potential Molecular Mechanisms of Their Anti-Inflammatory Activity

**DOI:** 10.3390/cimb47030145

**Published:** 2025-02-23

**Authors:** Zhaojia Li, Ran Meng, Wei Feng, Zhe Wu, Xuelin Lu, Xiuping Wang, Liangdan Sun

**Affiliations:** 1 Institute of Coastal Agriculture, Hebei Academy of Agriculture and Forestry Sciences, Tangshan 063299, China; tofriendzhaojia@163.com (Z.L.); fengwei522106@163.com (W.F.); wuzhe26@163.com (Z.W.); nkslxl@163.com (X.L.); 2 Faculty of Medicine, North China University of Science and Technology, Tangshan 063210, China; liangds@ncst.edu.cn

**Keywords:** dandelion, chlorogenic acid, caffeic acid, lipopolysaccharides, MD-2

## Abstract

This study explores the variation in the content of major phenolic acid components in dandelions from different regions, and the potential molecular mechanisms underlying their anti-inflammatory activity. High-performance liquid chromatography (HPLC) was employed to analyze dandelion leaves collected from four different regions in Hebei Province across eight harvest periods. The results indicated that chlorogenic acid had the highest content (0.334–1.963%), suggesting that this could be a key evaluation index for dandelion leaf harvesting. Further molecular docking and molecular dynamics simulations revealed that chlorogenic acid, caffeic acid, and chicoric acid could competitively bind to the key amino acid residues (e.g., PHE-151, ILE-117) of the MD-2 protein, thereby preventing the insertion of lipopolysaccharides (LPSs) and inhibiting the formation of the TLR4/MD-2 complex, which elucidates their potential anti-inflammatory mechanism. Moreover, environmental factors significantly influenced the accumulation of phenolic acids in dandelions, with temperature, precipitation, soil pH, and altitude showing correlations with the content variation of major phenolic acids. These findings provide a scientific basis for determining the optimal harvesting period of dandelion leaves, and offer new insights into the anti-inflammatory mechanisms of phenolic acids.

## 1. Introduction

The inflammatory response of cells is triggered by the stimulation of pro-inflammatory factors. Lipopolysaccharide (LPS), a common pro-inflammatory factor, is recognized by the pattern recognition receptor Toll-like receptor 4 (TLR4), and binds to its co-receptor myeloid differentiation protein 2 (MD-2) to form the LPS-TLR4/MD-2 complex. This complex subsequently undergoes dimerization with another LPS-TLR4/MD-2 complex, facilitating intracellular signal transduction. Ultimately, this process leads to the nuclear translocation of nuclear factor-kappa B (NF-κB), which initiates the transcription of target genes, thereby inducing the production of pro-inflammatory cytokines and triggering inflammation [1,2]. Studies have shown that the activation of the LPS-TLR4/MD-2 signaling pathway is considered a key factor in the onset of inflammation [3].

Dandelion (*Taraxacum mongolicum* Hand.-Mazz.) is a commonly used medicinal and edible herb that is rich in over 50 bioactive compounds, including phenolic acids, flavonoids, and sterols [4]. Among these, phenolic acids are considered the key active constituents contributing to the medicinal properties of dandelion. Modern pharmacological studies have confirmed that phenolic acids exhibit significant anti-inflammatory and immunoregulatory activities, both in vitro and in vivo [5,6,7,8,9,10,11,12]. These compounds exert their effects by modulating the expression of inflammatory factors such as TNF-α, IL-1β, IL-6, NO, caspase-3, and caspase-8 [5,13], as well as by blocking inflammation signaling pathways such as TLR4/NF-κB, thereby ameliorating LPS-induced inflammation [14,15]. However, due to the influence of complex environmental factors such as soil and climate, the content of major phenolic acid components in dandelion varies across different regions, leading to significant differences in its pharmacological effects [16,17]. This study focuses on dandelions from four different regions of Hebei Province, analyzing the major phenolic acid components, including chlorogenic acid, caffeic acid, and chicoric acid, using high-performance liquid chromatography (HPLC). A multi-region sampling strategy was employed to systematically study the variation in the content of these components. The sampling covered four representative geographical regions of Hebei Province, including plains, mountains, and coastal areas, with three sampling points in each region to ensure the representativeness and reliability of the data. Statistical methods, such as analysis of variance (ANOVA) and principal component analysis (PCA), were used to reveal the significant differences in the phenolic acid components of dandelion across regions and their correlations with environmental factors. On this basis, molecular docking and molecular dynamics simulations were further integrated to explore the anti-inflammatory mechanisms of dandelion phenolic acids. Molecular docking simulations of chlorogenic acid, caffeic acid, chicoric acid, and lipopolysaccharides (LPSs) with the MD-2 protein were conducted to calculate their binding free energy and identify key binding sites. Molecular dynamics simulations were used to assess the stability of the complexes, further confirming the potential anti-inflammatory activity of dandelion phenolic acids.

This study aims to evaluate the variation in the content of major phenolic acid components in dandelion across different regions through the integration of multi-region sampling and molecular docking techniques, and to explore the molecular mechanisms underlying its anti-inflammatory activity.

## 2. Sampling Area and Sample Information

**Figure 1:**
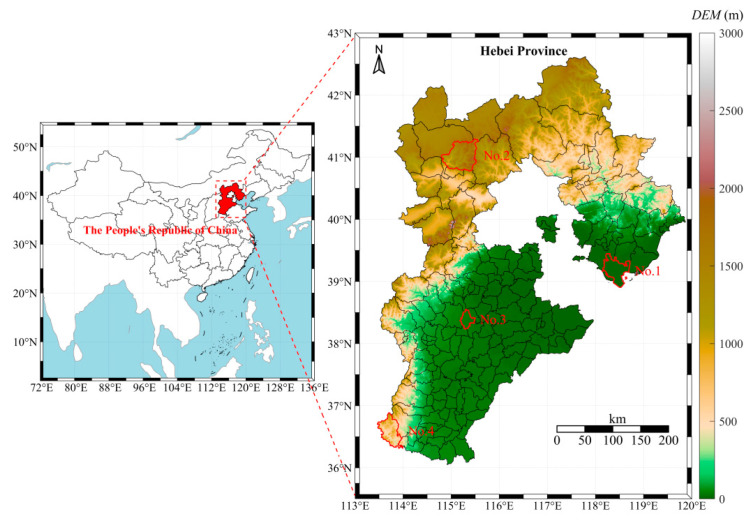
Sampling point distribution map. NO. 1 represents the sampling location in Caofeidian District. NO. 2 represents the sampling location in Chongli District. NO. 3 represents the sampling location in Anguo City. NO. 4 represents the sampling location in She County.

Hebei Province is situated between 36°05′–42°40′ N latitude and 113°27′–119°50′ E longitude, in North China, north of the Zhanghe River and adjacent to the Bohai Sea. The topography features higher elevations in the northwest and lower elevations in the southeast, with a general northwest-to-southeast incline. This region experiences a temperate continental monsoon climate, characterized by distinct seasonal variations. The annual average precipitation ranges from 300 to 800 mm, primarily concentrated in July and August. Dandelion leaf samples (labeled S1–S32) were collected from Caofeidian District in Tangshan, Chongli District in Zhangjiakou, Anguo City in Baoding, and Shexian County in Handan, as shown in Figure 1. Among them, Caofeidian District is a coastal plain dominated by coastal saline soil, with high salinity and alkaline pH. Chongli is a mountainous area with predominately brown soil and cinnamon soil, characterized by high soil organic matter content and neutral-to-slightly acidic pH. Anguo is a plain area with dominant fluvo-aquic soil, relatively high soil fertility, and neutral-to-slightly alkaline pH. Shexian is a mountainous and hilly region with primarily brown soil, high soil fertility, and neutral-to-slightly alkaline pH (Table 1). Sampling was conducted from 15 July 2023 to 30 October 2023, with samples collected every 15 days, resulting in a total of eight sampling events (Table 2).

## 3. Materials and Methods

### 3.1. Sampling Design and Sample Collection

Four representative geographical regions in Hebei Province were selected, including plains (Anguo), mountains (Chongli), coastal areas (Caofeidian), and hills (Shexian), representing different types of landforms. Three sampling points were established in each region to ensure coverage of the main habitat types within each area. The sampling period was standardized from July to October to ensure the consistent physiological status of the samples. At each sampling point, 10 healthy dandelion plants were randomly selected, and the aerial parts (including leaves and inflorescences) were collected, avoiding contamination from the root system. After collection, the samples were immediately placed in ice boxes for storage, and transported to the laboratory within 24 h. The collected dandelion samples were thoroughly washed with distilled water to remove surface dust and impurities. The samples were then dried at 60 °C until a constant weight was achieved, ground through an 80-mesh sieve, and stored in a desiccator for future use.

### 3.2. Extraction of Phenolic Acid Components

Precisely 0.1 g of the powdered dandelion sample was weighed and placed into a 25 mL volumetric flask, to which 5 mL of cellulase solution (Shanghai Macklin Biochemical Technology Co., Ltd., Shanghai, CN, concentration: 0.1%, pH 4) was added. After incubation at 60 °C in a water bath for 30 min, the solution was diluted to 25 mL with 70% methanol (Shanghai Macklin Biochemical Technology Co., Ltd., Shanghai, CN). Then, the mixture was subjected to ultrasonic treatment at 60 °C (power: 400 W, frequency: 40 kHz) for 120 min. Afterward, the solution was centrifuged (Sigma-Aldrich, State of Missouri, STL, USA) at 4000 rpm for 15 min, the supernatant was collected, and it was stored at 4 °C for future use [18].

### 3.3. Preparation of Mixed Standard Solution

Appropriate amounts of chlorogenic acid, caffeic acid, and chicoric acid standard compounds (Chengdu Desite Biological Technology Co., Ltd., Chengdu, CN)_ were accurately weighed, and each was placed into a 10 mL volumetric flask. To this, 70% methanol solution was added, followed by sonication to dissolve the compound, and then dilution to a certain volume to prepare standard stock solutions with concentrations of 0.260, 0.156, and 0.126 mg/mL, respectively. Next, a pipette was used to add 2.00 mL of each standard stock solution into separate 10 mL volumetric flasks. To each flask, 70% methanol was added and diluted to a certain volume, then shaken well to obtain a mixed standard solution containing 0.052 mg/mL of chlorogenic acid, 0.031 mg/mL of caffeic acid, and 0.025 mg/mL of chicoric acid.

### 3.4. Determination of Phenolic Acid Content

The sample solution was filtered through a 0.45 µm membrane using a syringe, and injected into the sample vial in preparation for HPLC analysis.

The analysis was conducted using an Agilent 1200 (Agilent Technologies, Inc. California, SC, USA) high-performance liquid chromatography system, coupled with a Mars ODS-AQ column (TMO, Waltham, MA, USA, 250 mm × 4.6 mm, 5 µm). The mobile phase comprised solvent A (0.2% phosphoric acid) and solvent B (methanol) in an equal ratio (1:1). Detection was carried out at a wavelength of 350 nm, with the column temperature meticulously maintained at 30 °C. The flow rate was precisely regulated at 1 mL/min, and the injection volume was set at 10 μL.

Standard curves were generated by establishing correlations between the reference compounds and their respective HPLC peak areas. Upon fitting, the resulting equations were as follows: Y_1_ = 0.164X_1_ + 0.929, Y_2_ = 0.390X_2_ + 0.814, Y_3_ = 0.353X_3_ − 1.251. In these equations, Y_1_, Y_2_, and Y_3_ correspond to the peak areas of chlorogenic acid, caffeic acid, and chicoric acid, respectively, while X_1_, X_2_, and X_3_ represent the concentrations of chlorogenic acid, caffeic acid, and chicoric acid in the dandelion samples, quantified (in μg/mL) within a validated detection range of 0–100 μg/mL.

### 3.5. Molecular Docking and Molecular Dynamics Simulation

#### 3.5.1. Bioinformatics Platforms and Software

Databases: PDB (Protein Data Bank, https://www.rcsb.org/search, accessed on 26 November 2023), PubChem (https://pubchem.ncbi.nlm.nih.gov, accessed on 26 November 2023).

Software: AutoDock Vina (version 1.2.3, TSRI, California, SD, USA). PyMol (version 2.5.5, DeLano Scientific LLC, New York, NY, USA). ADFR suite (version 1.0, TSRI, San Diego, CA, USA). Amber (version 22.0, UCSF, San Francisco, CA, USA).

#### 3.5.2. Molecular Docking of Chlorogenic Acid, Caffeic Acid, and Chicoric Acid with the LPS/TLR4/MD-2 Complex Crystal Structure

The crystal structure of the LPS/TLR4/MD-2 complex (PDB ID: 3FXI) was procured from the PDB database, while the three-dimensional structures of small molecules, including chlorogenic acid, caffeic acid, and chicoric acid, were retrieved from the PubChem database, and subsequently subjected to energy minimization using the MMFF94 force field. Utilizing PyMol 2.5.5, water molecules, salt ions, and small molecule contaminants were meticulously removed from the receptor protein. A docking box was then established, centered at the centroid of the ligand within the original crystal structure, with dimensions of 25 × 25 × 25 A^3^. Furthermore, all processed small molecules and the receptor protein were converted into the PDBQT format, requisite for docking with AutoDock Vina 1.2.3 using ADFRsuite 1.0. During the docking procedure, the exhaustiveness of the global search was set to 32, while all other parameters retained their default configurations. The docking conformation yielding the highest scoring output was regarded as the binding conformation. Finally, PyMol 2.5.5 was employed for a visual analysis of the interactions among chlorogenic acid, caffeic acid, chicoric acid, and the LPS/TLR4/MD-2 complex, encompassing the force field and amino acid sites.

#### 3.5.3. Molecular Dynamics Simulation

Molecular dynamics simulations were performed utilizing AMBER 22 software. Prior to the simulation, the system underwent a thorough energy optimization, incorporating 2500 steps of steepest descent, followed by an additional 2500 steps of conjugate gradient methods. The system was then subjected to a 200 ps heating phase, during which the temperature was gradually elevated from 0 K to 298.15 K. Maintaining this temperature, a 500 ps NVT (constant number of particles, volume, and temperature) ensemble simulation was conducted to further ensure the homogeneous distribution of solvent molecules within the solvent box. Following this, a 500 ps equilibrium simulation was executed under NPT (constant number of particles, pressure, and temperature) conditions for the entire system. Ultimately, the composite system underwent a 100 ns NPT ensemble simulation under periodic boundary conditions. Throughout the simulations, the non-bonded cutoff distance was established at 10 Å, and the Particle Mesh Ewald (PME) method was employed to calculate long-range electrostatic interactions. The SHAKE algorithm was applied to constrain hydrogen bond lengths, while the Langevin method was utilized for temperature regulation, with a collision frequency (γ) set at 2 ps−^1^. The system pressure was maintained at 1 atm, utilizing an integration time step of 2 fs, with trajectories saved every 10 ps for subsequent analysis.

### 3.6. Data Analysis

After the initial data organization using Microsoft Excel (version 16.0, MS, Redmond, WA, USA), one-way analysis of variance (ANOVA) was performed using SPSS software (version 26.0, SPSS Inc., Chicago, IL, USA), to compare significant differences in the phenolic acid content of dandelion samples from four different regions. Principal component analysis (PCA) was conducted using Origin software (version 10.0, OriginLab Corporation, Northampton, MA, USA) to analyze the phenolic acid content data, and both score plots and loading plots were generated. The distribution patterns of phenolic acid components in dandelions from different regions were examined. Pearson correlation analysis was employed to assess the relationship between phenolic acid content and environmental factors, with the significance level set at *p* < 0.05.

## 4. Results and Analysis

### 4.1. Correlation Analysis Between Environmental Factors and Phenolic Acid Components

**Figure 2:**
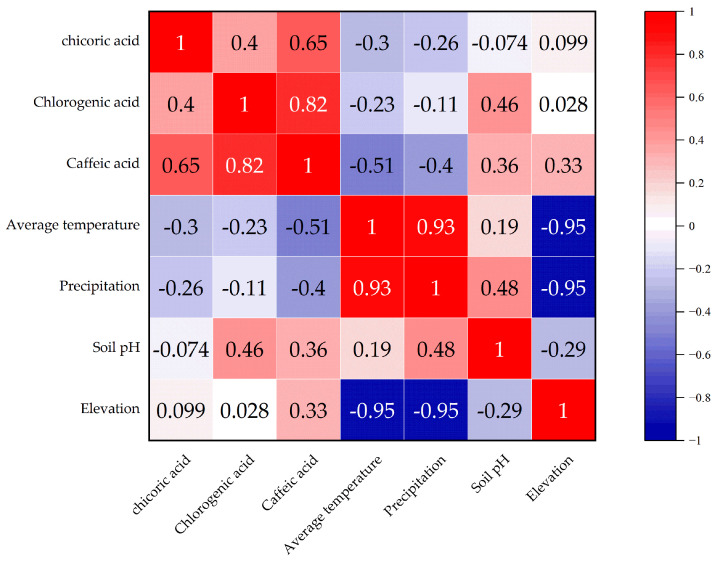
Correlation analysis between environmental factors and phenolic acid components.

As shown in Figure 2, the correlation between chlorogenic acid, chicoric acid, and caffeic acid demonstrates distinct patterns. The correlation between chlorogenic acid and caffeic acid is relatively high, while the correlation between chlorogenic acid and chicoric acid is relatively low. The correlation between chlorogenic acid and caffeic acid is the strongest, and is statistically significant (*p* < 0.05), suggesting that there may be a synergistic effect in the accumulation of these two phenolic acids in the samples.

The correlation analysis between environmental factors and phenolic acid components reveals that annual average temperature and precipitation are negatively correlated with caffeic acid, with no significance (*p* > 0.05), indicating that higher temperature and precipitation may suppress the accumulation of caffeic acid. Soil pH is positively correlated with chlorogenic acid and caffeic acid, but without statistical significance (*p* > 0.05), suggesting that soil pH may promote the accumulation of these two phenolic acids. Altitude is positively correlated with caffeic acid, but the correlation with chlorogenic acid and chicoric acid is weaker.

### 4.2. Variation in Chlorogenic Acid Content of Dandelions Across Different Regions

**Figure 3:**
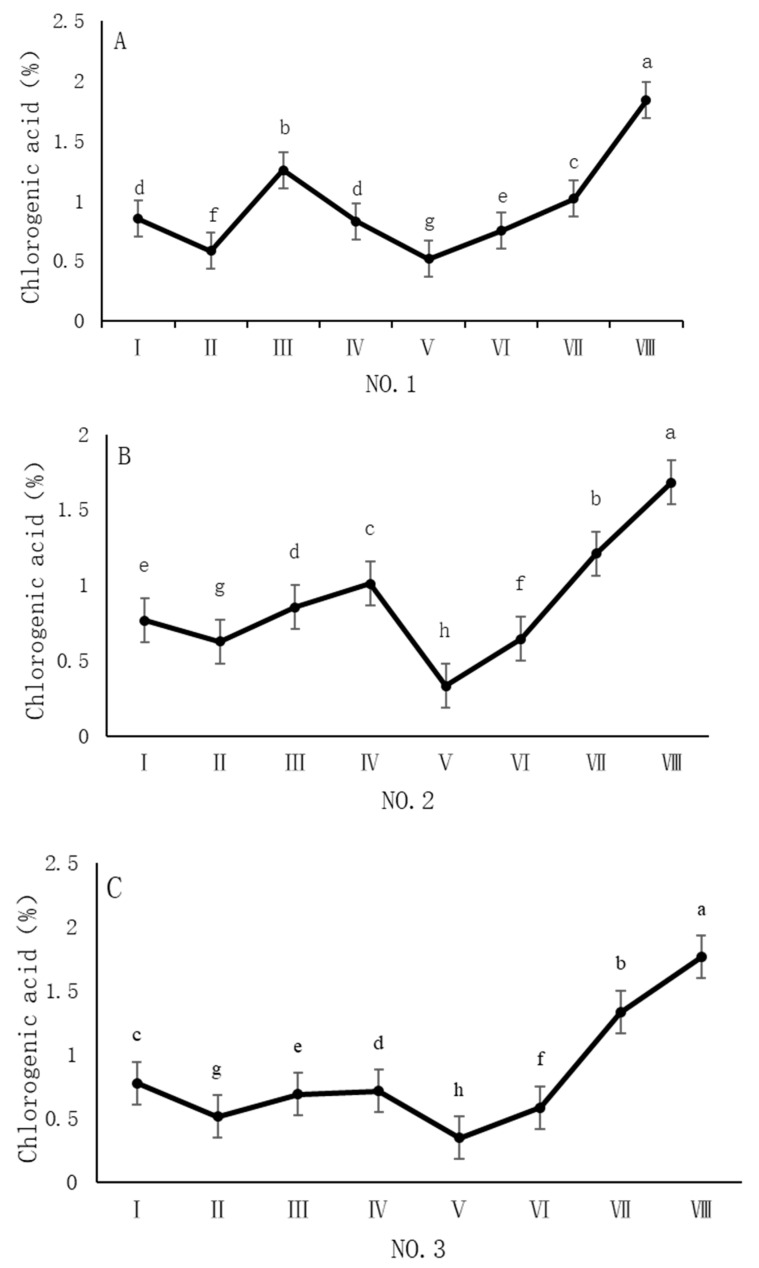

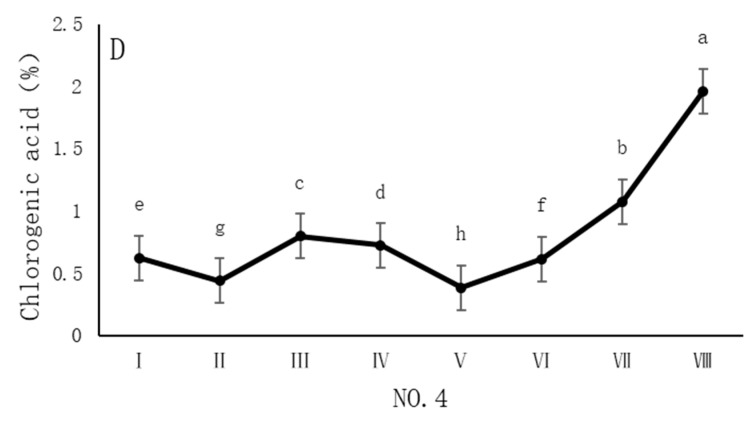
Variation in the chlorogenic acid content of dandelions across different regions. (**A**) Changes in the chlorogenic acid content of dandelion in NO.1 area. (**B**) Changes in the chlorogenic acid content of dandelion in NO.2 area. (**C**) Changes in the chlorogenic acid content of dandelion in NO.3 area. (**D**) Changes in the chlorogenic acid content of dandelion in NO.4 area. Note: different lowercase letters indicate significant differences (*p* < 0.05).

As shown in Figure 3, the dynamic changes in chlorogenic acid content in dandelion samples from different regions during the sampling period exhibited a significant “W”-shape trend. Specifically, the chlorogenic acid content in all four sampling regions reached its peak on 30 October, with values of 1.84%, 1.68%, 1.76%, and 1.96%, respectively. The chlorogenic acid content during this peak period was significantly different (*p* < 0.05) from the content in other sampling periods, indicating that the late October period is a critical time for chlorogenic acid accumulation.

### 4.3. Variation in Caffeic Acid Content of Dandelions Across Different Regions

**Figure 4:**
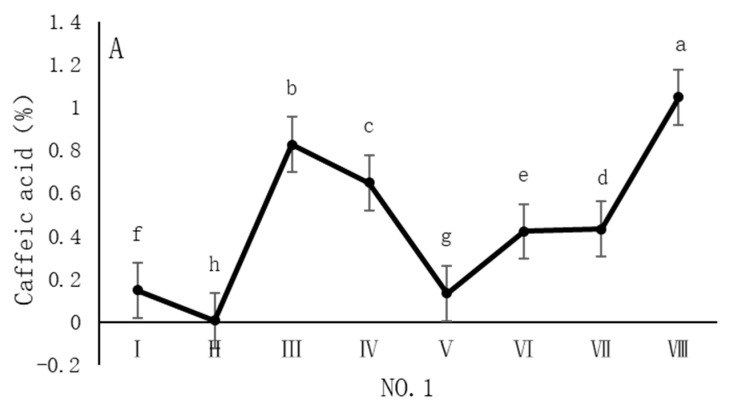

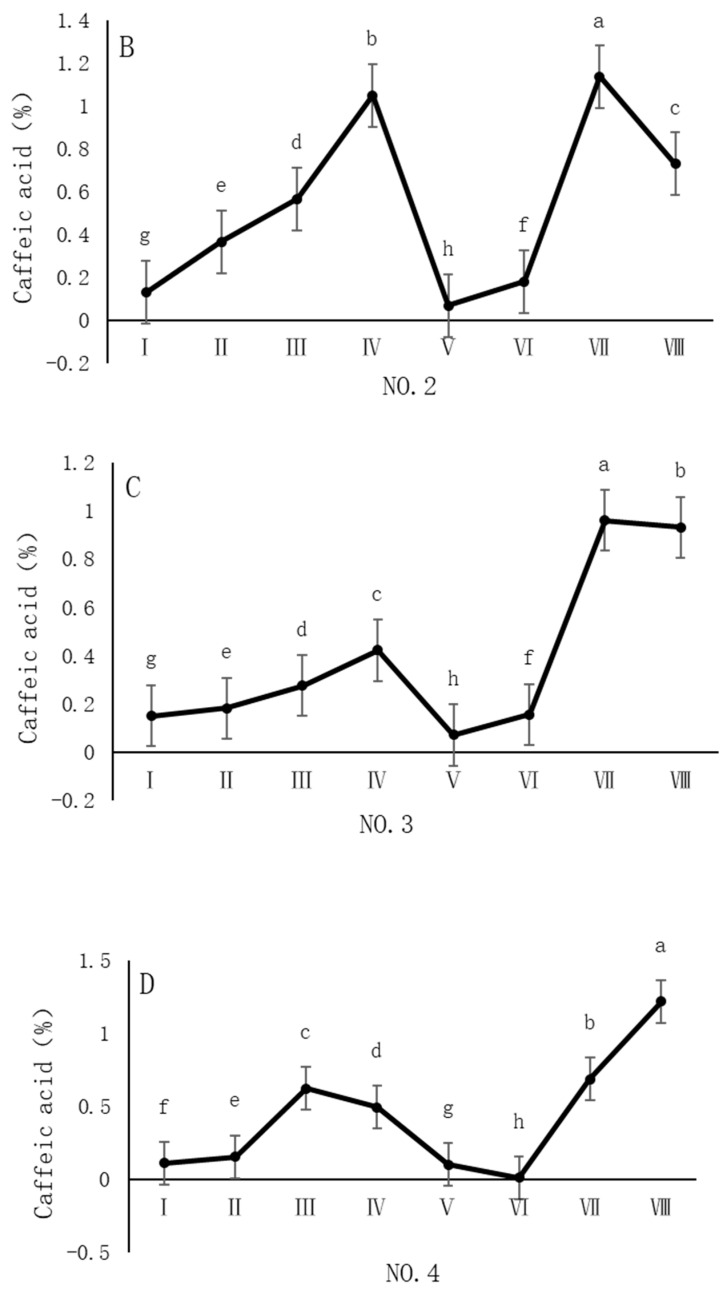
Variation in caffeic acid content of dandelions across different regions. (**A**) Changes in the caffeic acid content of dandelion in NO. 1 area. (**B**) Changes in the caffeic acid content of dandelion in NO. 2 area. (**C**) Changes in the caffeic acid content of dandelion in NO. 3 area. (**D**) Changes in the caffeic acid content of dandelion in NO. 4 area. Note: different lowercase letters indicate significant differences (*p* < 0.05).

As shown in Figure 4, the dynamic changes in caffeic acid content in dandelion samples during the sampling period exhibit distinct spatiotemporal distribution patterns. Specifically, the caffeic acid content in all sampling regions reached its peak in October, but the timing of the peak varied across regions. In the NO. 1 and NO. 4 sampling regions, the peak caffeic acid content occurred in late October, with concentrations of 1.05% and 1.22%, respectively. In contrast, the peak caffeic acid content in the NO. 2 and NO. 3 sampling regions occurred earlier, in mid-October, with concentrations of 1.13% and 0.96%, respectively. The caffeic acid content during the peak period was significantly different (*p* < 0.05) from the content in other sampling periods, indicating that October is a critical period for the biosynthesis and accumulation of caffeic acid in dandelions.

### 4.4. Variation in Chicoric Acid Content of Dandelions Across Different Regions

**Figure 5:**
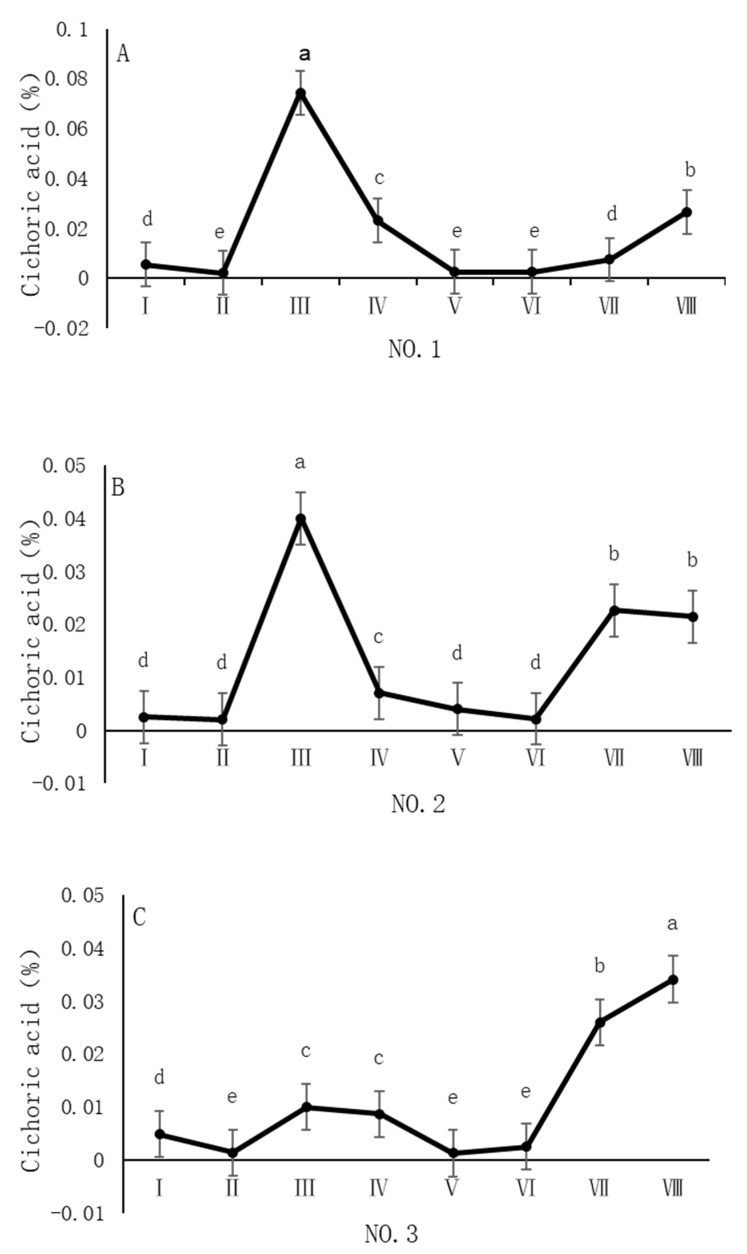

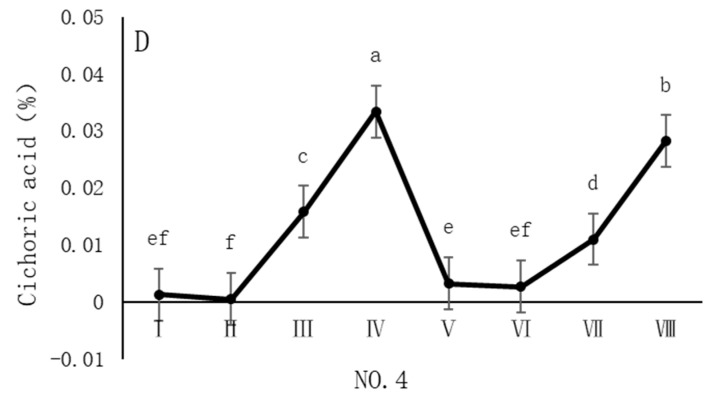
Variation in chicoric acid content of dandelions across different regions. (**A**) Changes in the chicoric acid content of dandelion in NO.1 area. (**B**) Changes in the chicoric acid content of dandelion in NO.2 area. (**C**) Changes in the chicoric acid content of dandelion in NO.3 area. (**D**) Changes in the chicoric acid content of dandelion in NO.4 area. Note: different lowercase letters indicate significant differences (*p* < 0.05).

As shown in Figure 5, the dynamic changes in chicoric acid content in dandelion samples across different regions during the sampling period exhibit a distinct “W”-shaped trend, with notable spatiotemporal heterogeneity. Specifically, in the NO. 1 and NO. 2 sampling regions, the chicoric acid content peaked in early August, with concentrations of 0.07% and 0.04%, respectively. In the NO. 4 sampling region, the peak occurred in late August, with a concentration of 0.03%. In the NO. 3 sampling region, the peak was delayed until late October, with a concentration of 0.03%. The chicoric acid content during these peak periods was significantly different (*p* < 0.05) from the content in other sampling periods, indicating that both August and October are critical periods for the biosynthesis and accumulation of chicoric acid.

### 4.5. Correlation Analysis of Major Phenolic Acid Components in Dandelions Across Different Regions

**Figure 6:**
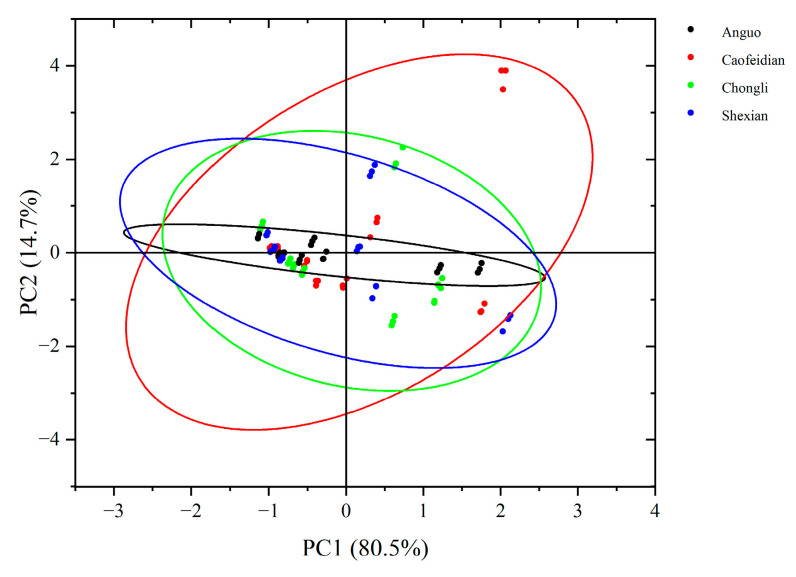
PCA score chart of dandelion phenolic acid composition. The black oval represent 95% Confidence Ellipse for Anguo. The red oval represent 95% Confidence Ellipse for Caofeidian. The green oval represent 95% Confidence Ellipse for Chongli. The blue oval represent 95% Confidence Ellipse for Shexian.

Based on the PCA score plot (Figure 6), it can be observed that PC1 explains 80.5% of the variance, effectively capturing the major variation in the samples, with a distinct spatial distribution. This suggests that geographical origin or environmental conditions significantly influence the characteristics of the dandelion samples. Specifically, the dandelion samples from Anguo and Shexian are closely grouped on PC1, indicating high similarity in their main variance features. In contrast, the dandelion samples from Cao Feidian and Chongli are more dispersed, suggesting distinct differences in their characteristics.

**Figure 7:**
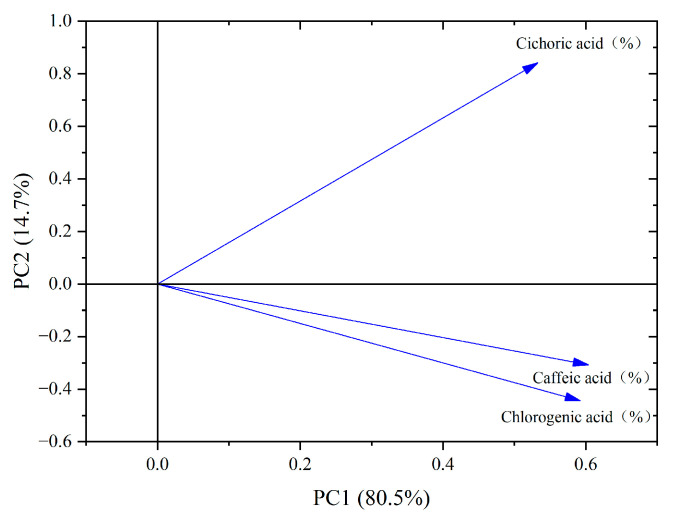
PCA loading diagram of phenolic acid components in dandelion.

From the PCA loading plot (Figure 7), it can be seen that chlorogenic acid, caffeic acid, and chicoric acid exhibit high positive loadings on PC1, indicating their significant contribution to the first principal component. On PC2, chicoric acid also shows a high positive loading, indicating its considerable contribution to the second principal component. Meanwhile, chlorogenic acid and caffeic acid have relatively lower loadings on PC2, suggesting their minimal contribution to this component. Additionally, the correlation between chlorogenic acid and caffeic acid is the strongest among the three phenolic acids, highlighting their close relationship in the data.

### 4.6. Visualization of the Interaction Between Chlorogenic Acid, Caffeic Acid, Chicoric Acid, LPS, and MD-2

**Figure 8:**
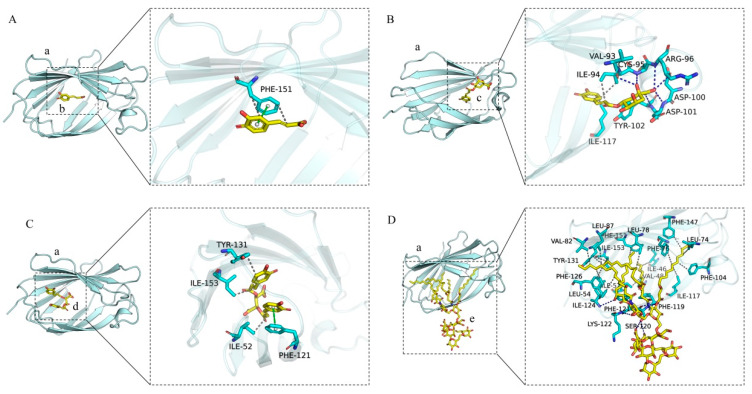
Interactions of chlorogenic acid, caffeic acid, chicoric acid, and LPS with MD-2. (**A**) The interaction diagram between caffeic_acid and the MD2 protein. (**B**) The interaction diagram between chlorogenic acid and the MD2 protein. (**C**) The interaction diagram between chicoric acid and the MD2 protein. (**D**) The interaction diagram between LPS and the MD2 protein. The left image shows the overall view, while the right image presents a detailed view. (a) MD-2 protein. (b) caffeic_acid small molecule. (c) chlorogenic acid small molecule. (d) chicoric acid small molecule. (e) LPS small molecule. The gray dashed lines represent hydrophobic interactions, the green dashed lines indicate pi-pi conjugation interactions, and the blue lines denote hydrogen bond interactions. The cyan sticks represent the amino acid residues.

As depicted in Figure 8A–D, the left panel provides a comprehensive overview, while the right panel delivers a detailed perspective. In these representations, (a) corresponds to the MD-2 protein, whereas (b), (c), (d), and (e) represent caffeic acid, chlorogenic acid, chicoric acid, and LPS, respectively, all of which are capable of integrating into the hydrophobic pocket of the MD-2 subunit. Caffeic acid establishes π-π stacking interactions and hydrophobic contacts with the amino acid residue PHE-151 of the MD-2 protein. Chlorogenic acid forms hydrogen bonds with the amino acid residues VAL-93, CYS-95, TYR-102, ASP-101, ASP-100, and ARG-96, while simultaneously engaging in hydrophobic interactions with ILE-117 and ILE-94. Chicoric acid interacts with the PHE-121 residue of the MD-2 protein through π-π stacking, and forms hydrophobic interactions with TYR-131, ILE-153, and ILE-52. LPS interacts with MD-2 by forming hydrogen bonds with the amino acid residues LYS-122, ILE-124, and SER-120, and engages in extensive hydrophobic interactions with a multitude of residues, including LEU-78, LEU-74, LEU-54, PHE-147, PHE-126, VAL-82, VAL-48, ILE-117, TYR-131, ILE-52, PHE-151, ILE-124, PHE-76, LEU-87, PHE-104, PHE-119, ILE-46, PHE-121, and ILE-153. A comparative analysis of the interaction sites among caffeic acid, chlorogenic acid, chicoric acid, and MD-2, in conjunction with the interaction sites of LPS with MD-2, elucidates that the amino acid residues PHE-151, ILE-117, PHE-121, TYR-131, ILE-153, and ILE-52 within the MD-2 protein serve as shared interaction sites for caffeic acid, chlorogenic acid, chicoric acid, and LPS.

### 4.7. Molecular Dynamics Simulations of Chlorogenic Acid, Caffeic Acid, Chicoric Acid, LPS, and MD-2

**Figure 9:**
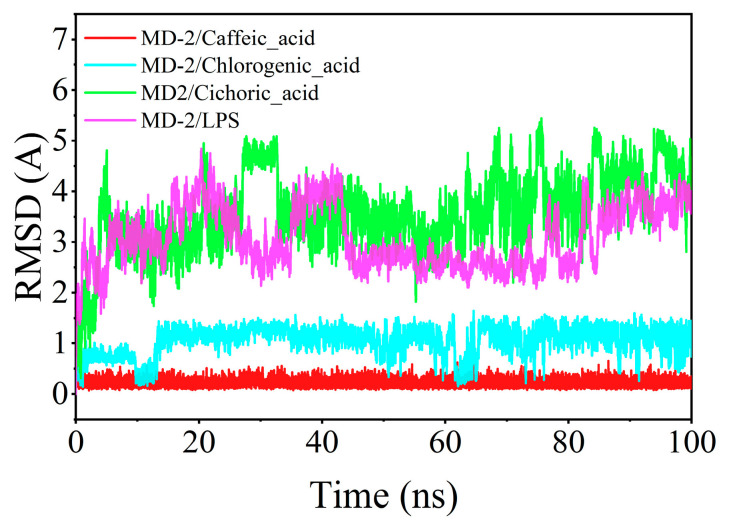
Time-dependent changes in the root mean square deviation (RMSD) of ligands during molecular dynamics simulations.

The root mean square deviation (RMSD) serves as a critical indicator of ligand mobility throughout the binding process. Elevated RMSD values, accompanied by increased fluctuations, signify more vigorous motion, while lower values denote enhanced stability. The RMSD fluctuations observed for chlorogenic acid, caffeic acid, and MD-2 during their interaction remained below 2 Å, indicating a more stable integration within the binding pocket compared to LPS. In contrast, chicoric acid demonstrated a comparatively diminished binding stability with MD-2 (Figure 9).

**Figure 10:**
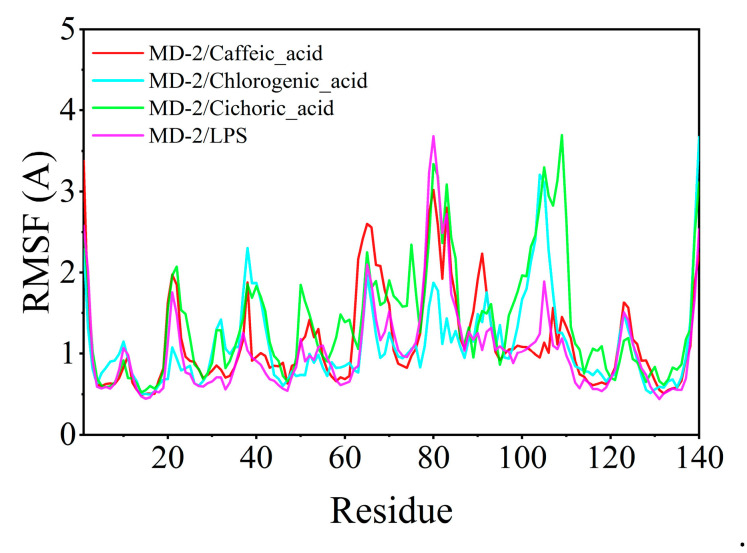
Root mean square fluctuation (RMSF) calculated from molecular dynamics simulation trajectories.

The root mean square fluctuation (RMSF) serves to elucidate the flexibility of the protein during molecular dynamics simulations. Typically, the binding of a ligand to a protein results in decreased protein flexibility, thereby enhancing the stability of the protein and promoting its enzymatic activity. Excluding specific localized regions, the RMSF of the protein consistently remained within 2 Å. The RMSF values of MD-2 when complexed with chlorogenic acid, caffeic acid, and chicoric acid were analogous to those observed with LPS, suggesting that chlorogenic acid, caffeic acid, and chicoric acid exhibit binding effects on the protein comparable to those of LPS (Figure 10).

## 5. Discussion

Temperature and precipitation are key environmental factors influencing the accumulation of plant secondary metabolites. Studies have shown that temperature can regulate the expression levels of several key enzymes in the phenylpropanoid metabolism pathway, thereby affecting the biosynthesis of phenolic acids [19]. Under optimal temperature conditions, the activities of enzymes such as phenylalanine ammonia-lyase (PAL) and cinnamate-4-hydroxylase (C4H) are typically high, promoting the synthesis of phenolic acids [20]. However, high temperatures may accelerate the degradation of phenolic acids while inhibiting the expression of key enzymes in their biosynthetic pathway, resulting in reduced accumulation of compounds such as chlorogenic acid and caffeic acid [21]. Additionally, our study found that higher temperatures decrease the accumulation of caffeic acid, while low temperatures favor its synthesis. This phenomenon may be related to the plant’s enhancement of secondary metabolic pathways under low-temperature stress to increase antioxidant capacity and adaptability. Furthermore, increased precipitation may dilute the accumulation of phenolic acids and affect the availability of soil nutrients, thus indirectly influencing the synthesis of secondary metabolites [22]. Soil pH affects plant absorption of mineral elements, thereby regulating the accumulation patterns of secondary metabolites [23]. In this study, soil pH showed a positive correlation with the levels of chlorogenic acid and caffeic acid, suggesting that alkaline environments may promote the accumulation of these two phenolic acids. Previous studies have indicated that higher soil pH usually enhances the availability of minerals such as calcium and magnesium, which may promote the synthesis of phenolic acids by regulating the activities of key enzymes like PAL and C4H. Higher pH levels may also reduce the toxicity of certain metal ions in the soil, enabling plants to maintain normal metabolic activity and improving the efficiency of secondary metabolic pathways [24]. Simultaneously, elevation showed a positive correlation with caffeic acid accumulation, while its correlation with chlorogenic acid and chicoric acid was weaker. This trend may be related to the increased light intensity, temperature fluctuations, and ultraviolet (UV) radiation levels at high altitudes. High-altitude environments typically enhance plant secondary metabolic activity to allow them to cope with environmental stress, especially with increased UV radiation, which can induce the expression of key enzymes such as PAL, thereby promoting the synthesis of phenolic acids such as caffeic acid [25]. Moreover, elevation may indirectly regulate the accumulation of secondary metabolites by influencing soil temperature and humidity, nutrient availability, and vegetation growth. Therefore, future studies could combine environmental monitoring data from different altitudes to further elucidate the specific regulatory mechanisms of elevation on phenolic acid accumulation.

Our analysis reveals distinct correlation patterns in the accumulation of chlorogenic acid, caffeic acid, and chicoric acid in *Taraxacum officinale* samples. Chlorogenic acid showed a higher correlation with caffeic acid (*p* < 0.05), while the correlation between chlorogenic acid and chicoric acid was weaker. Both chlorogenic acid and caffeic acid are phenylpropanoid compounds primarily synthesized through the phenylalanine pathway [26], with caffeic acid being a direct precursor of chlorogenic acid. Therefore, they may accumulate synergistically, resulting in a higher correlation between the two. In contrast, the biosynthesis pathway of chicoric acid is more complex, involving further hydroxylation and esterification of chlorogenic acid or caffeic acid, and is regulated by specific enzymes [27]. The accumulation of chicoric acid is also closely related to environmental stresses, such as UV radiation and pathogen infection, which may cause its accumulation pattern to be influenced by various environmental factors [20], leading to a weaker correlation between chlorogenic acid and chicoric acid. Furthermore, the physiological functions of chlorogenic acid, caffeic acid, and chicoric acid differ in the plant, which may further influence their accumulation patterns. Chlorogenic acid is involved in antioxidant defense, UV protection, and disease resistance [28], while caffeic acid also plays a crucial role in antioxidant defense [29]. Chicoric acid, in addition to antioxidant and antimicrobial properties, may be associated with secondary metabolism regulation and plant growth and development [30]. Thus, the differences in their accumulation in various growth environments may reflect their distinct physiological functions and environmental adaptation strategies. In terms of temporal dynamics, the accumulation of chlorogenic acid, caffeic acid, and chicoric acid exhibited different trends. The accumulation of chlorogenic acid and chicoric acid showed a significant “W” shape, with chlorogenic acid peaking in late October, caffeic acid reaching a phase peak in October, and chicoric acid peaking in both August and October. These variations are closely related to the plant’s growth stages, environmental conditions, and secondary metabolic regulatory mechanisms. The accumulation of chlorogenic acid is closely associated with its physiological functions in the plant and its storage and degradation dynamics, likely serving antioxidant and disease resistance functions in early growth stages, and enhancing stress resistance in later stages [21]. The accumulation of caffeic acid shows notable spatiotemporal distribution characteristics, which are potentially closely related to environmental factors such as temperature, light, and soil nutrients [22], with regional differences. Chicoric acid also exhibited a “W”-shaped accumulation pattern, and its synthesis is regulated by UV radiation and environmental stress, particularly under high levels of light or stressful conditions [25], leading to a significant increase in its content. PCA demonstrated distribution differences between samples from different sampling regions, reflecting the impact mechanisms of environmental factors on the accumulation of phenolic acids. The scores of the samples from Anguo and Shexian were similar, possibly due to similar climatic and soil conditions, while the scores of the samples from Caofeidian and Chongli were more dispersed, potentially influenced by differing altitudes and soil conditions. The low temperatures and strong UV radiation in Chongli may result in distinct secondary metabolic pathways compared to low-altitude areas, thus affecting the accumulation patterns of phenolic acids. Moreover, the variation in the accumulation of caffeic acid and chicoric acid between different sampling regions indicates that the accumulation patterns of these compounds are closely related to local environmental stresses, such as drought and low temperature. Plants may adjust the phenylpropanoid metabolism pathway to synthesize antioxidant secondary metabolites, enhancing their ability to adapt to environmental conditions.

Recent studies have shown that plant-derived phenolic acid compounds possess significant anti-inflammatory effects. For instance, Salvianolic acid B (SalB) can significantly reduce the expression levels of TLR4, Myd88, and NF-κB proteins in the kidney tissues of rats with renal injury [31], while total phenolic acids from *Saussurea involucrata* can inhibit the phosphorylation of TAK1, c-Jun, and IRF3, as well as the expression of NF-κB p65, thereby reducing the inflammatory response in RAW264.7 cells induced by LPS [32]. Both SalB and PSI exhibit their anti-inflammatory effects through the LPS-TLR4/MD-2-NF-κB signaling pathway, suggesting that TLR4/MD-2 proteins are important targets for LPS-induced inflammation. Therefore, targeting TLR4/MD-2 for the identification of anti-inflammatory drugs holds significant research value. Does *Taraxacum officinale* contain phenolic acid compounds that can bind to TLR4/MD-2 and exert anti-inflammatory effects? In this study, we targeted TLR4/MD-2 and used molecular docking and molecular dynamics simulation methods to virtually screen and analyze the binding modes of the main phenolic acids in *T. officinale*—chlorogenic acid, caffeic acid, and chicoric acid. The results showed that all three compounds could fit into the hydrophobic pocket of the MD-2 protein and interact with key amino acid residues (such as PHE-151, ILE-117, etc.). Furthermore, by comparing the binding modes of LPS with the MD-2 protein, we found that chlorogenic acid, caffeic acid, and chicoric acid overlap with LPS at the binding site, suggesting that they may inhibit LPS insertion by competitively binding to the MD-2 protein, thereby suppressing the activation of the TLR4/MD-2 signaling pathway and exerting anti-inflammatory effects. Molecular docking results further revealed that chlorogenic acid, caffeic acid, and chicoric acid can all stabilize their binding to the MD-2 protein through hydrogen bonding, hydrophobic interactions, and π-π conjugation. Molecular dynamics simulations were further conducted to assess the stability of these phenolic acid-MD-2 protein complexes. The RMSD analysis indicated that chlorogenic acid and caffeic acid showed RMSD fluctuations below 2 Å in the MD-2 protein binding pocket, which was even better than that seen for LPS, suggesting that they can stably bind to the MD-2 protein. RMSF analysis showed that the RMSF values of the MD-2 protein upon binding with chlorogenic acid and caffeic acid were similar to those observed when binding with LPS, indicating that chlorogenic acid and caffeic acid may interfere with the activation of the TLR4/MD-2 signaling pathway through a binding mode similar to that of LPS. In contrast, chicoric acid exhibited larger RMSD fluctuations and poorer binding stability, suggesting that its stability of conformation to the MD-2 protein may not be as favorable as that of chlorogenic acid and caffeic acid.

## 6. Conclusions

Although this study reveals the influence patterns of environmental factors on the accumulation of phenolic acids in Taraxacum officinale and explores its potential molecular mechanisms, several limitations remain. Firstly, the study is based on data from specific sampling areas and time periods; therefore, future research should include long-term, large-scale monitoring to validate the generalizability of the findings. Secondly, the accumulation of phenolic acids is not only influenced by environmental factors, but may also be regulated by gene expression. Thus, future studies combining transcriptomics and metabolomics approaches could further explore the regulatory mechanisms underlying phenolic acid synthesis in T. officinale. Additionally, molecular docking and dynamics simulation results suggest that chlorogenic acid and caffeic acid may exhibit strong pharmacological activity, which should be further investigated through cellular and animal experiments to validate their biological effects.

## Figures and Tables

**Table 1:** Environmental data of sampling locations.

Region	Average Annual Temperature (°C)	Precipitation (mm)	Soil pH	Altitude (m)
Caofeidian	11.5	650	8.0	5
Chongli	6.5	400	6.5	1200
Anguo	12	600	7.8	30
Shexian	12.3	580	7.2	500

**Table 2:** Information of dandelion samples.

Place of Origin	Number	Place of Origin	Number	Growth Period	Harvesting Time
(NO. 1) Caofeidian District, Tangshan City, Hebei Province	S1	(NO. 3) Anguo City, Baoding City, Hebei Province	S17	I	15 July 2023
	S2		S18	II	30 July 2023
	S3		S19	III	15 August 2023
	S4		S20	IV	30 August 2023
	S5		S21	V	15 September 2023
	S6		S22	VI	30 September 2023
	S7		S23	VII	15 October 2023
	S8		S24	VIII	30 October 2023
(NO. 2) Chongli District, Zhangjiakou City, Hebei Province	S9	(NO. 4) She County, Handan City, Hebei Province	S25	I	15 July 2023
	S10		S26	II	30 July 2023
	S11		S27	III	15 August 2023
	S12		S28	IV	30 August 2023
	S13		S29	V	15 September 2023
	S14		S30	VI	30 September 2023
	S15		S31	VII	15 October 2023
	S16		S32	VIII	30 October 2023

## Data Availability

Data derived from the current study can be provided to readers upon request.
